# How public health insurance expansion affects healthcare utilizations in middle and low-income households: an observational study from national cross-section surveys in Vietnam

**DOI:** 10.1186/s12889-023-15500-6

**Published:** 2023-03-31

**Authors:** Chi M. Nguyen, Mai P. Nguyen, Lan D. P. Luc

**Affiliations:** 1 Indiana University School of Medicine, Indiana University – Purdue University Indianapolis, Indianapolis, USA; 2 Queensland University of Technology, Brisbane City, QLD Australia; 3 Department of Medical Services Administration, Ministry of Health, Hanoi, Vietnam; 4 Macquarie Business School, Macquarie University, Sydney, Australia

**Keywords:** Public health insurance expansion, Middle-income, Low-income, Vietnam, Doubly-robust difference-in-differences

## Abstract

Public health insurance (PHI) has been implemented with different levels of participation in many countries, from voluntary to mandatory. In Vietnam, a law amendment made PHI compulsory nationwide in 2015 with a tolerance phase allowing people a flexible time to enroll. This study aims to examine mechanisms under which the amendment affected the enrollment, healthcare utilization, and out-of-pocket (OOP) expenditures by middle- and low-income households in this transitioning process.

Using the biennial Vietnam Household Living Standard Surveys, the study applied the doubly robust difference-in-differences approach to compare outcomes in the post-amendment period from the 2016 survey with those in the pre-amendment period from the 2014 survey. The approach inheriting advantages from its predecessors, i.e., the difference-in-differences and the augmented inverse-probability weighting methods, can mitigate possible biases in policy evaluations due to the changes within the group and between groups over time in the cross-section observational study.

The results showed health insurance expansion with extensive subsidies in premiums and medical coverage for persons other than the full-time employed, young children or elderly members in the family, significantly increased enrollments in the middle- and low-income groups by 9% and 8%, respectively. The number of visits for PHI-eligible services also increased, approximately 0.5 more visit per person in the middle-income and 1 more visit per person in the low-income. The amendment, however, so far did not show any significant effect on reducing OOP payments, neither for the low nor the middle-income groups. To further expand PHI coverage and financial protections, policymakers should focus on improving public health facilities, contracting PHI to more accredited private health providers, and motivating the high-income group’s enrollments.

**Supplementary Information:**

The online version contains supplementary material available at 10.1186/s12889-023-15500-6.

## Introduction

Expansion of public health insurance (PHI) has been a central focus to achieve universal health coverage (UHC) in many low- and middle-income countries (LMICs). The purpose of this focus is to ensure that everyone can have affordable access to needed and quality health services without financial hardship [1]. A recent systematic review showed that public health insurance policies in LMICs acquired quite different results in health access and financial protection as each country implemented PHI at different levels of enforcement, from voluntary to obligatory participation [2]. However, little was known about the immediate effects of transitioning from a partially to a fully compulsory PHI in those countries. This study is to investigate those effects regulated by a law amendment in Vietnam.

Vietnam is one of the LMICs that committed to adopting UHC as a national strategy in the early 2000s and set a goal of achieving UHC by 2030 [3]. Ever since the country has been making continuous efforts toward its goal by massively expanding PHI coverage with a gradually increasing level of obligation [4]. To increase the enrollment toward more than 95% of the population by 2030, Vietnam passed an amendment of the Law on Health Insurance in 2014 that officially designated PHI as compulsory nationwide. The amendment expanded PHI eligibility, provided more incentives and subsidies in both premiums and medical coverages, and mandated employers’ contribution to PHI premiums for their employees. It also provided a tolerance period that no personal penalty for not being enrolled in PHI was imposed at this stage [5].

Along the timeline of fundamental reforms of the health insurance policy over the last decade, many studies have been conducted to evaluate the effects of PHI on health access and utilization by specific groups of populations in Vietnam. For example, some studies focused on children aged 6 and under [6–8] while others examined the vulnerable households [9, 10], using data up to the year 2012, i.e., the period before this amendment became effective.

To our best knowledge, this study is the first to examine the impacts of compulsory PHI expansion stipulated by this amendment – the most important reform of the past decade in Vietnam – on the middle- and low-income households that accounted for 90% of the Vietnamese population. We employed a doubly robust difference-in-differences (DR-DID) approach proposed by Sant’Anna and Zhao (2020) [11] to evaluate these impacts on enrollments, utilization and out-of-pocket health expenditures.

### Vietnam’s PHI background

The PHI was first established in 1992 with two types of schemes, compulsory and voluntary. The compulsory scheme was initially offered to only civil servants and pensioners [12]. During the period from 1992 to 2008, PHI gradually expanded the compulsory scheme for other groups including people with social welfare allowance, veterans, poor households, ethnic minorities, older adults over 80 years of age, and people with disability or working capacity loss [13] resulting in an increase in the insurance coverage from 5.4% in 1992 to more than 40% in 2008 [4, 14].

The promulgation of the Law on Health Insurance effective in July 2009 officially laid the first legislative foundation for enrolling every resident into PHI. In addition to expanding compulsory PHI to all workers/employees with labor contracts, the law provided more generous financial supports in both premiums and medical expenses to the poor and the vulnerable with a fully subsidized scheme for children aged 6 or younger (zero premiums and 100% medical expense coverage). The PHI coverage increased from 44% in 2008 to 71% in 2014 [14]. However, PHI was still voluntary for a rather large part of the population such as informal laborers, dependents and other household members [15].

**Fig. 1:**
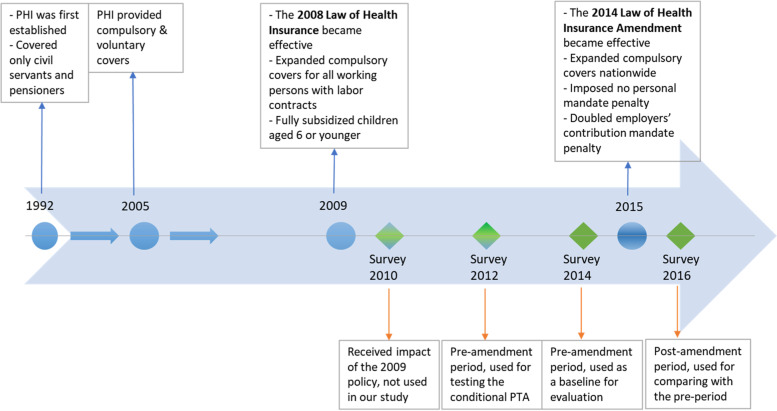
The time-line of changes in public health insurance policies, the PHI law and its amendment

An important amendment of the Law was passed in 2014 and officially made PHI compulsory nationwide from January 2015. It encouraged the enrollments of the hard-to-reach populations, covering part-time laborers and dependent household members. Particularly, the amendment expanded the eligibility for premium subsidies, lowers the premium for each additional household member who was not the first PHI enrollee and neither working nor receiving government subsidies. Moreover, the amendment doubled the penalty on employers for failing to contribute two-third of PHI premiums for their employees in due time but did not impose a personal mandate penalty for not being enrolled in PHI [5]. Consequently, the insurance coverage rate reached close to 82% in 2016 [16]. See Fig. 1 for the timeline of these changes.

In terms of medical expenses covering prescriptions and expenses for two eligible services, i.e., illness care, and pregnancy and maternity care,^1^ the amendment considerably expanded groups of beneficiaries who received 100% coverage, including the vulnerable population such as poor households, ethnic minorities living in socio-economic disadvantage areas, pensioners, receivers of certain kinds of social and disability allowances. Near-poor households were also eligible for 95% expense coverage instead of 80% as in the earlier period [5]. However, most of these medical expenses were fully covered by PHI only if people already sought care from public health providers.

Additionally, the PHI reform facilitated and encouraged health insurance use in public health facilities by relaxing some bypassing health-facility restrictions. In the past, to get full expense coverage enrollees were required to seek care from their registered primary care providers. However, starting from 2016 they can use PHI to receive care from any public care provider in their same residential province [5, 17].

Besides PHI, there are currently 18 commercial life insurance companies in Vietnam [18], governed by Vietnam commercial law instead of the law on health insurance. People can enroll voluntarily in these commercial health insurance programs to supplement their healthcare plans of which services are either only partially or not covered by PHI. In general, PHI enrollees get reimbursement for their eligible health expenses if they visit public providers and a few number of PHI contracting private providers.

## Methods

### Data

The data were extracted from three waves of the Vietnam Household Living Standard Surveys (VHLSS) that was conducted by the Vietnamese General Statistics Office to monitor and supervise the living standards of households nationwide. The survey was first implemented in 1992 with technical support from World Bank, then improved with more representative sampling schemes from 2002, and has been repeatedly conducted once every two years ever since. This repeated cross-section survey was acquired via face-to-face interviews with randomized households^2^ at the commune level across 63 provinces and centrally-run cities and rural/urban areas in Vietnam, where communes were primary sampling units (PSU) with each containing 1,600 households on average [19, 20].

As the study goal is to evaluate the impacts of the amendment that became effective in 2015, we used the survey waves 2014 and 2016 for comparing the pre and post-amendment outcomes. The number of respondents in the 2014 and 2016 waves were 36,057 and 35,787 persons from 9,396 and 9,399 households, respectively. The questionnaires asked about health information of all household members within the past 12 months, including health insurance types, sickness/injury encounters, reasons for a visit, payments for each visit, drug and other health-related expenditures (see Appendix 1 – Examples of Healthcare Questionnaires for more details). The data were then linked with household income, demographics, education, and employment information in VHLSS. Additionally, the earlier wave in 2012 was used for testing the robustness of our model’s identification and reported in Appendix 2.

### Outcome measures and explanatory variables

Four outcomes were used to evaluate the amendment effects on the middle and low-income groups, i.e., the percentage of PHI enrollments, the number of visits for services that are eligible for PHI benefits, the OOP payments for these PHI-eligible services, and total health expenditure in the pre and post-amendment. The services eligible for PHI benefits included maternal care and illness care. The total expenditure tallied health insurance premiums, charges for all health visits, drug expenses, medical assistive-device expenses, and subtracting healthcare allowances if applicable.

To explain these outcomes, two types of health-related covariates were considered. The first type, personal health status in the past 12 months, was used as a covariate for the PHI enrollment outcome. It was coded as an indicator, taking value 1 if having normal health and 0 otherwise, where the normal health status was assigned to respondents who reported neither encountering any severe injury nor paying any health visit for illness nor maternity care. The second type was the two medical services covered by PHI, including illness care and maternity care, for explaining the number of PHI-eligible services, the OOP for PHI services, and the total expenditure. These services were also coded as indicators, 1 for using it, and 0 otherwise.

Personal employment status was classified in three exclusive categories: being employed (wage earners), self-employed (independent/small contractors, proprietors in either farm or non-farm sectors), and neither of the above. Moreover, as it is well known that household wealth plays an important role in modelling socio-economic behaviors in risk-pooling strategy, especially in insurance and public investments [21], and serves as a key social determinant of health, we included the natural logarithm of the household income^3^ to account for each group’s income elasticity.

In addition, broad-age categories (aged from 0 – 19, from 20 – 39, from 40 – 59, and 60 years or older), genders (female vs. male), ethnicity groups (minor vs. major), marital statuses (married vs. not married), the number of household members (household size), education level of the household head, and the household’s location (urban vs. rural areas) were also included to adjust for other sociodemographic personal and household characteristics.

### Characteristics of different income groups before and after the PHI amendment

This study investigated three income household groups, i.e., the high-income, the middle-income, and the low-income, focusing on the amendment’s impacts on the middle- and the low-income ones. The high-income group were the top-10%-income households in each survey wave. The low-income group was the poor and near-poor households with incomes below or near the poverty line defined by the Vietnam Ministry of Labor, War Invalids and Social Affairs in 2016 [22]. The middle-income group included the rest of the households.

Table 1 provides a summary of each group's characteristics and their percentage changes over the two periods. The VHLSS housing weights and PSU clusters were applied to obtain the nationally representative estimates. The middle-income and the low-income had more significant changes across the 2 waves than the high-income group. All groups showed aging trends with increasing percentage of people older than 60 years. Certain characteristics were observed to be unique to each group, notably employment status, education of the head of the household, and residence location among others. The low-income had the highest percentages of either unemployed or self-employed including mostly independent contractors, small proprietors, but the lowest percentages of wage-earners, compared to the high-income (41.9% unemployed compared to 32.6% and 38.9%, and 40.7% self-employed compared to 31.8% and 30.7% in the high-income and middle-income, respectively). Big differences in income growth across these groups were observed, at 18%, 13%, and 5% in the high-, middle-, and low-income, respectively. The head of household with a college or higher degree accounted for less than 1% in the low-income group while that was more than 30% in the high-income. Nearly 90% of the low-income resided in a rural area, but only 35% of the high-income did.

**Table 1:** Characteristics of households in the pre and post-PHI amendment periods (the VHLSS housing weights and Primary Sampling Units clusters were applied)

	**High-income group**		**Middle-income group**		**Low-income group**	
	*2014*	*2016*	*2014*	*2016*	*2014*	*2016*
**Variable**	*Pre*	*Post*	*Pre*	*Post*	*Pre*	*Post*
	***n* = 2,814**	***n* = 2,546**	***n* = 24,932**	***n* = 27,103**	***n* = 8,311**	***n* = 6,138**
**Age 0 -19, % (s.e.)**	17.5 (0.8)	17.1 (0.9)	26.5 (0.3)	26.2 (0.3)	32.9 (0.7)	32.7 (0.8)
**Age 20—39, % (s.e.)**	30.2 (1.1)	28.3 (1.3)	27.6 (0.3)	25.4 (0.3)	24.6 (0.6)	23.5 (0.7)
**Age 40—59, % (s.e.)**	39.3 (1.2)	39.0 (1.4)	30.3 (0.4)	30.7 (0.4)	20.9 (0.7)	20.9 (0.8)
**Age 60 + , % (s.e.)**	13 (1.1)	15.6 (1.2)	15.6 (0.4)	17.7 (0.4)	21.6 (1.1)	23 (1.3)
**Female, % (s.e.)**	51.7 (0.9)	50.2 (1.0)	51.9 (0.3)	52.1 (0.3)	54.1 (0.7)	55 (0.8)
**Ethnic minority, %**	4.0 (0.7)	6.0 (1.0)	8.3 (0.4)	9.8 (0.4)	39.8 (1.5)	48.6 (1.7)
**Married, % (s.e.)**	57.9 (1.2)	58.1 (1.3)	52.4 (0.4)	52 (0.4)	45.8 (0.9)	45.2 (1.0)
**Employment status**						
Being-employed, % (s.e.)	37.1 (1.4)	35.5 (1.6)	31.1 (0.4)	30.4 (0.4)	18.7 (0.6)	17.4 (0.7)
Self-employed, % (s.e.)	28.7 (1.4)	31.8 (1.4)	30.6 (0.4)	30.7 (0.4)	39.2 (1.0)	40.7 (1.1)
Not employed, % (s.e.)	34.2 (1.2)	32.6 (1.2)	38.3 (0.4)	38.9 (0.4)	42.1 (0.9)	41.9 (0.9)
**Income/person/month (logarithm), mean (s.e.)**	8.8 (0.01)	9.0 (0.01)	7.7 (0.01)	7.8 (0.01)	6.48 (0.01)	6.53 (0.02)
**Education of head of household**						
Primary school/lower, % (s.e.)	21.6 (1.5)	22.3 (1.8)	43.8 (0.7)	44.9 (0.7)	73.6 (1.2)	74 (1.3)
Secondary/high school, % (s.e.)	46.7 (2.0)	47.5 (2.1)	50.3 (0.7)	48.2 (0.7)	26.3 (1.2)	25.8 (1.3)
College or higher, % (s.e.)	31.7 (1.9)	30.2 (2.0)	5.9 (0.3)	6.9 (0.4)	0.1 (0.1)	0.3 (0.1)
**Rural residence, % (s.e.)**	34.9 (1.8)	35.1 (2.0)	68.9 (0.5)	68.5 (0.4)	87.1 (0.9)	88.9 (0.9)
**Household size, mean (s.e.)**	3.3 (0.06)	3.3 (0.06)	3.8 (0.02)	3.8 (0.02)	4.0 (0.05)	4.0 (0.07)

**Fig. 2:**
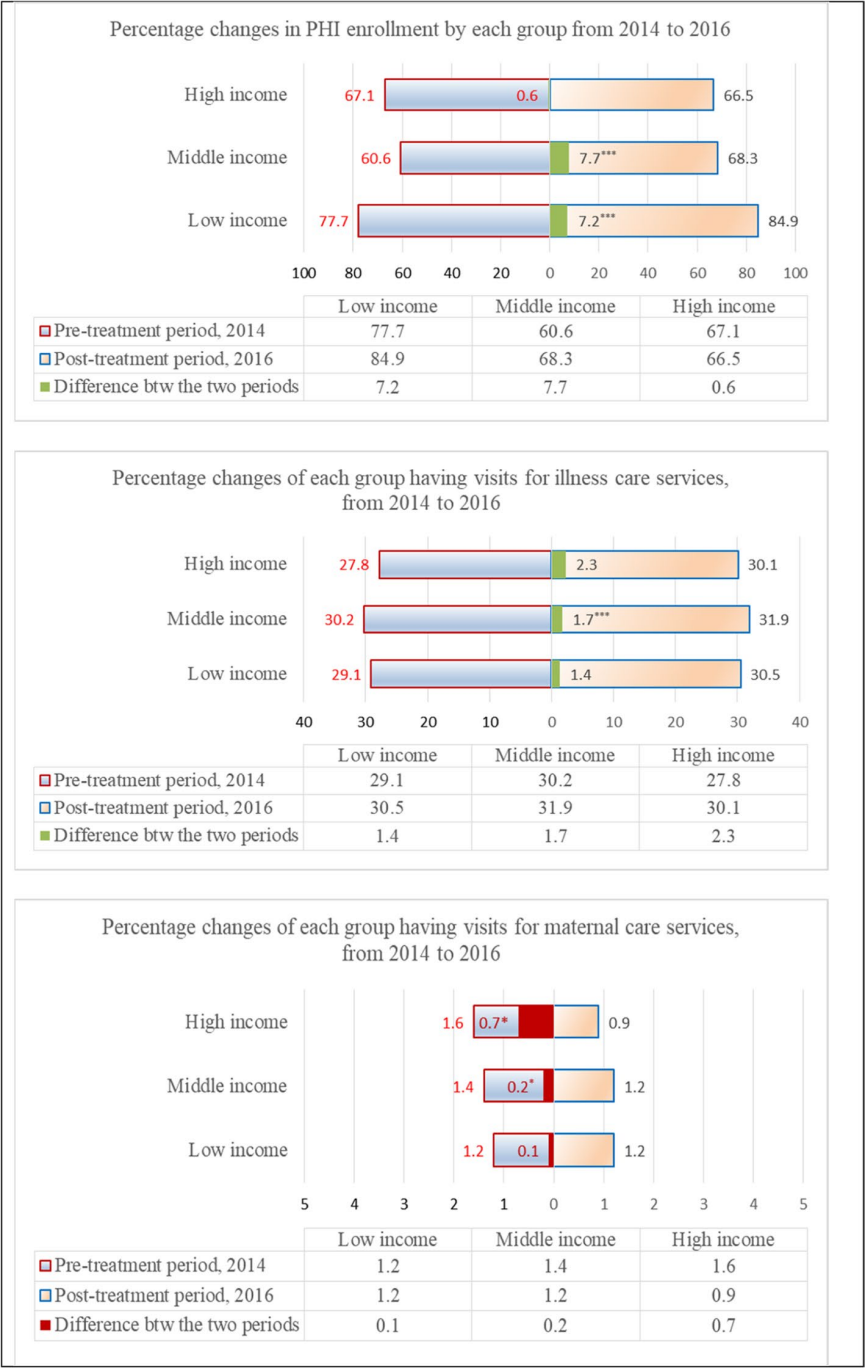
Percentage of PHI enrollments and having at least an eligible service, and their differences over the two periods in each group

Taking into account these differences, it is anticipated that the PHI amendment would affect the three groups differently. The high-income didn't show any significant change in PHI enrollments in the post-amendment period, and slightly decreased by 0.6%. PHI enrollments by the middle and the low income groups, however, significantly increased by 7.7% and 7.2% respectively. The middle-income had their illness visits significantly increased by 1.7% (Fig. 2). It was possible that the additional benefits of the amendment emphasizing financial incentives more than fines would barely affect the high-income but mostly motivate the low- and middle-income groups.

### Statistical analysis: Doubly robust difference-in-differences approach

This study adopted the difference-in-differences (DID) design with inverse-probability weighing scheme to examine the causal effect of the expansion of compulsory health insurance on health service use. The study faced two common challenges of public health policy research where randomized controlled trials are unable to conduct [23]. The first was to construct a suitable control group for comparison under the context that the PHI amendment became effective to all groups at the same time. As the high-income showed more stable patterns in PHI enrollments and health utilization in the pre and post-treatment periods than the other two groups (see Table 1), this group was the least responsive to the change, thereby served as the control group.

Doing so, however, encountered the second challenge of policy research. In this repeated cross-sectional surveys, selection bias possibly existed across groups due to the groups’ differences in many characteristics. Also selection bias across time that happened when the groups changed in composition between the two periods, i.e., households can move from low- to middle-income, or from middle- to high-income group and vice versa as their income changed. Their heterogeneous dissimilarities, especially those that are also endogenously related to their trends across time such as employment status, income growth or other socio-economic conditions, could contribute more to invalidating the common trend assumption in the conventional and unweighted DID design, resulting in misleading inferences [24].

Therefore, our study employed the doubly robust difference-in-differences approach (DR-DID) proposed by Sant’Anna and Zhao [11]. The DR-DID inherited advantages from three predecessors, the Heckman bias-correction, the DID and the augmented inverse propensity weighting (AIPW) methods. It combined the bias-corrected outcome regression [25] and the DID inverse probability-weighted estimators [26], where the Heckman’s regression accounts for the unique outcome mechanism of the control group in each period, and the Abadie’s inverse probability weighing addresses the bias due to the changes in group composition across time.

In DR-DID, the outcome evolutions within each group were predicted separately in pre- and post-treatment period and the corresponding difference over time within the group were measured. Next, the difference between control and treated groups were compared and estimated.^4^ In very step, inverse propensity score weights were properly applied to the control’s outcome evolutions to achieve a consistent estimate of an average treatment effect on the treated group (ATT) when either the outcome mechanism in the control group or the probability of being in the treated group is correctly specified.

Particularly, first a standard treatment design of two periods and two groups was adopted. Denote *t* the treatment periods where *t* = 0 in the pre-treatment period, and *t* = 1 in the post-treatment period. Denote *d* the status of a group where *d* = 0 for the control group, *d* = 1 for the treated group; ${Y}_{i,d,t}$ the outcome of a person *i* in a group *d* at time *t*, having a set of characteristics ${X}_{i,d,t}$; and $n$ the total sample size. The ATT measured the expected difference in potential outcomes between the group receiving treatment ${Y}_{i,\mathrm{1,1}}\left(1\right)$ and its counterfactual group who hypothetically didn’t receive treatment at the time ${Y}_{i,\mathrm{1,1}}\left(0\right)$, i.e.,

1 $$\tau = {\mathbb{E}}\left({Y}_{i,\mathrm{1,1}}\left(1\right) -{Y}_{i,\mathrm{1,1}}\left(0\right)|{d}_{i}= 1\right)$$

Following the DR-DID approach, then the conditional expected outcome of the control group, denoted as ${{\mathbb{E}}\left({Y}_{i,0,t}|X,{d}_{i}=0\right)= \widehat{\mu }}_{0,t}(X)$, was estimated *separately in each period t* (*t* = 0, 1) that allowed for tracking the group’s unique evolution in the pre and post-treatment period. This captured a full set of effects of explanatory factors X on the outcomes Y in the control group changing from the pre to post-treatment period that may present the group’s unique behaviors, making the outcome evolutions more time-specific and not averaged out over time as the way popularly used in the AIPW method. In our study, a logistic model was used to estimate the likelihood of PHI enrollment, and a negative binomial regression^5^ for the expected numbers of PHI-eligible visits. Then, linear models for the expected OOP for PHI-eligible services, and for the tallied expenditures in natural logarithm forms were used. All these models belong to the class of generalized linear models presented in natural log forms [27]. Additionally, outcome mechanism of the middle- and low-income in each period were provided.

Second, a consistent weighting scheme was estimated using pooled data. For the control group, the weights, denoted as ${\widehat{\omega }}_{i,0,t},$ were the scaled inversed propensity score ${\widehat{\omega }}_{i,0,t}(X)=\frac{\widehat{\pi }\left({X}_{i,0,t}\right) 1(T=t)/(1-\widehat{\pi }{\left(X\right.}_{i,0,t}))}{{\mathbb{E}}\{\widehat{\pi }\left({X}_{i,0,t}\right) 1\left(T=t\right)/(1-\widehat{\pi }\left({X}_{i,0,t}\right.))\}}$ where $\widehat{\pi }\left(X\right)$ was the estimated probability of the control group being in the treated group, and the indicator 1(.) equal to 1 if its argument was true and 0 otherwise. For the treated group, the weights were the scaled empirical ones ${\widehat{\omega }}_{i,1,t}=\frac{\left({d}_{i}=1\right) 1(T=t)}{{\mathbb{E}}\left[\left({d}_{i}=1\right) 1(T=t)\right]}$. Hereafter, subscript *i* is dropped for notation convenience. The study implemented pairwise comparisons, i.e., the middle- versus the high-income, and the low- versus the high-income groups. A logistic regression adjusted for personal and household characteristics were employed in each pair to predict the propensity scores of the control group falling into the treated groups and their inverses $\{{\widehat{\omega }}_{\mathrm{0,0}}, {\widehat{\omega }}_{\mathrm{0,1}}\}$.

And third, the following ${\widehat{\tau }}^{DR.DID}$ by Sant’Anna and Zhao [11] to estimate the ATT on the middle- and low-income groups was employed,

2 $${\widehat{\tau }}^{DR.RC}={\mathbb{E}}_{n}\left\{({\widehat{\omega }}_{1}-{\widehat{\omega }}_{0})(Y-{\widehat{\mu }}_{0,Y})\right\}$$

where ${\mathbb{E}}_{n}\left(Z\right)= \frac{1}{n}\sum_{i=1}^{n}{Z}_{i}$ the empirical expectation, ${\widehat{\omega }}_{1}= {\widehat{\omega }}_{\mathrm{1,1}}-{\widehat{\omega }}_{\mathrm{1,0}}$, and $Y=T\cdot Y_1+(1-T)\cdot Y_0$ and ${\widehat\mu}_{0,Y}=T\cdot{\widehat\mu}_{0,1}+(1-T)\cdot{\widehat\mu}_{0,0}$ of which ${\widehat{\mu }}_{0,t}$ was the expected outcomes of the control group conditional on *t*. This estimand did not subtract the conditional outcome of the treated group, following discussions in [28, 29]. Under the conditional parallel trend assumption (PTA) and the standard assumptions of program evaluations, it was showed that ${\widehat{\tau }}^{DR.RC}$ was consistent and doubly robust. A specification test for the conditional PTA was also provided using the data in two pre-amendment survey waves, 2012 and 2014. In the absence of the amendment, the changes in outcomes between these 2 pre-waves should not be significantly different from zeros. The test results were supportive of the PTA (see Appendix 2 – Conditional PTA tests).

In addition to the full sample of survey waves, we further examined the outcomes in 3 subsamples including (i) only the PHI beneficiaries, (ii) the persons having at least one visit for PHI-eligible services regardless of enrolling into PHI or not, and (iii) the PHI beneficiaries with at least one visit for PHI-eligible services. All analysis were performed in R version 4.0 × and Stata-SE17.

## Results

### The outcome mechanisms of each household group

#### Preference for public health insurance enrollment

Relationships between PHI enrollments and individual/household characteristics in pre- and post-treatment periods were analyzed for the high-, the middle-, and the low-income households and presented in Table 2. Certain relations were similar in tendency but different in magnitude in the 3 groups. Factors associated with higher likelihoods of enrolling PHI included minor ethnicity, not-employed status, and higher education attainment of the head of household. The association of minor ethnicity with PHI enrollments were strongest in the low-income group with estimated effects of 1.79 in 2014 and 1.36 in 2016, while in the middle- and high-income groups they were 1.30 and 0.93 in 2014, and 0.91 and 0.49 in 2016, respectively. The effects of non-working status on the PHI enrollment were of 0.46 and 1.6 in the high-income, 1.08 and 1.09 in the middle-income, and 0.88 and 0.72 in the low-income group in 2014 and 2016, respectively. Higher education seemed to induce higher enrollments, though not significantly in the low-income group.

**Table 2:** The mechanisms of factors associated with PHI enrollments in each household group

	**High-income group**		**Middle-income**		**Low-income**	
**Variable**	*Pre—2014*	*Post—2016*	*Pre—2014*	*Post—2016*	*Pre—2014*	*Post -2016*
	*Coef (se)*	*Coef (se)*	*Coef (se)*	*Coef (se)*	*Coef (se)*	*Coef (se)*
**Normal health**	-0.44 (0.15)^c^	-0.2 (0.15)	-0.58 (0.05)^c^	-0.35 (0.05)^c^	-0.50 (0.11)^c^	-0.24 (0.13)^a^
***Age group (reference: Age 0—19)***						
Age 20—39	-1.99 (0.25)^c^	-1.88 (0.32)^c^	-1.63 (0.08)^c^	-1.62 (0.08)^c^	-0.75 (0.17)^c^	-0.43 (0.21)^b^
Age 40—59	-1.17 (0.30)^c^	-1.08 (0.37)^c^	-1.18 (0.08)^c^	-1.14 (0.09)^c^	-0.72 (0.20)^c^	-0.51 (0.22)^b^
Age 60 +	-0.41 (0.35)	-0.52 (0.43)	-0.26 (0.10)^c^	-0.29 (0.10)^c^	-0.15 (0.20)	-0.14 (0.25)
**Female**	0.05 (0.09)	-0.004 (0.12)	-0.02 (0.03)	-0.03 (0.03)	-0.02 (0.06)	-0.04 (0.08)
**Minor ethnicity**	0.93 (0.44)^b^	0.49 (0.27)^a^	1.30 (0.13)^c^	0.91 (0.11)^c^	1.79 (0.17)^c^	1.36 (0.22)^c^
**Married**	0.32 (0.18)	0.26 (0.20)	-0.12 (0.06)^b^	-0.03 (0.06)	-0.31 (0.14)^b^	-0.32 (0.17)^a^
***Employment status (reference: Being-employed)***						
Self-employed	-0.65 (0.18)^c^	0.19 (0.16)	-0.05 (0.06)	0.10 (0.05)^b^	0.03 (0.11)	0.23 (0.13)^a^
Not-employed	0.46 (0.22)^b^	1.60 (0.27)^c^	1.08 (0.08)^c^	1.09 (0.08)^c^	0.88 (0.16)^c^	0.72 (0.18)^c^
***Education of the household head (reference: primary school or lower)***						
Secondary or high school	0.27 (0.17)	0.53 (0.19)^c^	0.45 (0.05)^c^	0.50 (0.05)^c^	-0.08 (0.13)	0.02 (0.16)
College or higher	0.50 (0.22)^b^	0.98 (0.21)^c^	1.79 (0.14)^c^	1.42 (0.12)^c^	na	0.42 (1.24)
**Household income** (in natural log)	-0.15 (0.20)	-0.03 (0.22)	0.14 (0.07)^b^	-0.08 (0.06)	-0.78 (0.19)^c^	0.03 (0.13)
**Household size**	0.05 (0.05)	0.06 (0.05)	-0.01 (0.02)	0.02 (0.02)	-0.07 (0.03)^b^	-0.09 (0.04)^b^
**Rural residence**	0.08 (0.17)	0.20 (0.17)	-0.001 (0.06)	0.08 (0.06)	0.12 (0.15)	0.58 (0.21)^c^
**constant**	1.61 (0.39)^c^	0.56 (0.44)	1.16 (0.12)^c^	1.09 (0.12)^c^	1.59 (0.27)^c^	1.31 (0.35)^c^
**Pseudo R**^**2**^	0.13	0.18	0.20	0.17	0.17	0.10
**Obs**	2,814	2,546	24,932	27,103	8,311	6,138

^a, b, c^represented 10%, 5% and 1% significance levels, respectively

On the other hand, factors that significantly associated with less likelihood of PHI enrollments were the normal health status and the ages. Persons who did not have any health issue in the past 12 months were less likely to enroll PHI than those who did (Coef = -0.44 and -0.20 for the high-income group, -0.58 and -0.35 for the middle-income group, and -0.50 and -0.24 for the low-income group in 2014 and 2016, respectively). Compared to the ages from 0–19, older age seemed negatively associated with PHI enrollments, especially the ages from 20 to 39 (Coef = -1.99 and -1.88 for the high-income, -1.63 and -1.62 for the middle-income, and -0.75 and -0.43 for the low-income group in 2014 and 2016, respectively).

The results also found that certain factors changed their significant influences over the two periods, and these changes were unique to each group. Particularly, the self-employed persons in the high-income group were less likely enrolled into PHI in the post-treatment period while those in the middle- and low-income groups were more likely. Changes in the household income of the high-income groups did not affect the preference for PHI enrollment in both periods. Yet, household income changes of the middle- and low-income groups switched its influence from negatively affecting the PHI enrollment in the pre-treatment period to insignificantly in the post-treatment period. Household size also discouraged only the low-income group from enrolling PHI (Coef = -0.07 and -0.09 in 2014 and 2016).

#### Number of PHI-eligible visits and out-of-pocket payment

As PHI only reimbursed illness and maternity care services, healthcare visits due to either illness or maternity or both were counted, and their related out-of-pocket payment (OOP) correspondingly estimated to aim at evaluating direct impacts of the PHI policy changes on its eligible-service utilizations in the latter section. Factors associated with the occurrence of eligible services were presented in the clustered columns “Number of visits”, and those with the related OOP were in the columns “Out-of-pocket payment” of Table 3.

**Table 3:** The mechanisms of factors associated with number of PHI-eligible visits and out-of-pocket payment in each household group

	**Number of PHI-eligible visits**						**Out-of-pocket payment for PHI-eligible services**					
	**High-income**		**Middle-income**		**Low-income**		**High-income**		**Middle-income**		**Low-income**	
	*2014*	*2016*	*2014*	*2016*	*2014*	*2016*	*2014*	*2016*	*2014*	*2016*	*2014*	*2016*
*Variable*	*Coef (se)*	*Coef (se)*	*Coef (se)*	*Coef (se)*	*Coef (se)*	*Coef (se)*	*Coef (se)*	*Coef (se)*	*Coef (se)*	*Coef (se)*	*Coef (se)*	*Coef (se)*
**Illness**	4.24 (0.20)^c^	4.43 (0.25)^c^	4.23 (0.07)^c^	4.27 (0.07)^c^	4.56 (0.12)^c^	4.54 (0.15)^c^	6.48 (0.13)^c^	6.47 (0.11)^c^	5.97 (0.04)^c^	5.95 (0.04)^c^	5.34 (0.09)^c^	4.83 (0.11)^c^
***Age group (reference: Age 0—19)***												
Age 20—39	0.89 (0.26)^c^	0.3 (0.19)	0.35 (0.07)^c^	0.54 (0.07)^c^	0.35 (0.13)^c^	0.46 (0.14)^c^	0.32 (0.11)^c^	0.41 (0.12)^c^	0.42 (0.05)^c^	0.50 (0.05)^c^	0.07 (0.10)	0.13 (0.11)
Age 40—59	-0.46 (0.35)	0.37 (0.17)^b^	0.03 (0.07)	0.26 (0.07)^c^	0.12 (0.12)	0.49 (0.13)^c^	-0.11 (0.18)	0.27 (0.14)^a^	0.18 (0.05)^c^	0.22 (0.05)^c^	-0.1 (0.10)	-0.02 (0.12)
Age 60 +	0.2 (0.23)	0.57 (0.14)^c^	0.35 (0.07)^c^	0.48 (0.05)^c^	0.26 (0.10)^c^	0.43 (0.12)^c^	0.01 (0.17)	0.44 (0.16)^c^	0.23 (0.05)^c^	0.26 (0.05)^c^	-0.01 (0.09)	0.23 (0.11)^b^
**Female**	0.74 (0.17)^c^	0.31 (0.09)^c^	0.32 (0.04)^c^	0.32 (0.03)^c^	0.29 (0.06)^c^	0.14 (0.06)^b^	0.22 (0.08)^c^	0.11 (0.07)	0.21 (0.02)^c^	0.19 (0.02)^c^	0.17 (0.04)^c^	0.06 (0.05)
**Minor ethnicity**	-0.41 (0.19)^b^	-0.25 (0.21)	-0.09 (0.09)	-0.04 (0.08)	-0.38 (0.08)^c^	-0.39 (0.08)^c^	-0.38 (0.28)	-0.08 (0.18)	-0.13 (0.05)^b^	-0.08 (0.05)	-0.17 (0.05)^c^	-0.26 (0.08)^c^
**Married**	0.83 (0.27)^c^	0.29 (0.13)^b^	0.35 (0.05)^c^	0.28 (0.05)^c^	0.21 (0.10)^b^	0.13 (0.09)	0.50 (0.15)^c^	0.25 (0.11)^b^	0.29 (0.04)^c^	0.26 (0.04)^c^	0.37 (0.09)^c^	0.42 (0.11)^c^
***Employment status (reference: Being-employed)***												
Self-employed	-0.06 (0.25)	-0.16 (0.13)	-0.03 (0.05)	0.02 (0.05)	-0.16 (0.10)	-0.13 (0.12)	0.03 (0.13)	-0.05 (0.09)	0.07 (0.03)^b^	0.05 (0.03)	0.18 (0.06)^c^	0.11 (0.08)
Not employed	-0.01 (0.22)	0.30 (0.16)^a^	0.19 (0.07)^c^	0.30 (0.06)^c^	0.12 (0.11)	0.21 (0.13)	0.28 (0.13)^b^	0.34 (0.12)^c^	0.22 (0.05)^c^	0.31 (0.05)^c^	0.09 (0.10)	0.15 (0.11)
***Education of the household head (reference: primary school or lower)***												
Secondary or high school	-0.04 (0.15)	-0.27 (0.11)^b^	-0.12 (0.04)^c^	-0.14 (0.04)^c^	-0.14 (0.08)^a^	-0.20 (0.09)^b^	0.17 (0.10)	-0.04 (0.10)	0.12 (0.02)^c^	0.08 (0.03)^c^	0.1 (0.06)	0.11 (0.08)
College or higher	0.04 (0.18)	-0.44 (0.13)^c^	-0.09 (0.08)	-0.13 (0.07)^a^	-0.16 (0.14)	-0.39 (0.14)^c^	-0.13 (0.12)	-0.12 (0.12)	0.05 (0.06)	-0.05 (0.05)	-0.04 (0.16)	1.92 (1.09)
**Household income (in ln)**	0.57 (0.24)^b^	0.14 (0.22)	-0.06 (0.06)	-0.04 (0.04)	0.19 (0.11)	0.06 (0.05)	0.18 (0.11)	0.14 (0.11)	0.09 (0.03)^c^	0.15 (0.03)^c^	0.37 (0.09)^c^	0.07 (0.06)
**Household size**	-0.04 (0.06)	0.05 (0.03)	0.02 (0.01)^b^	0.02 (0.01)^b^	0.02 (0.02)	0.02 (0.02)	0.02 (0.04)	0.03 (0.03)	-0.01 (0.01)	0.01 (0.01)	0.04 (0.01)^c^	0.05 (0.02)^c^
**Rural residence**	-0.02 (0.16)	-0.06 (0.11)	-0.07 (0.05)	-0.05 (0.04)	0.004 (0.10)	0.03 (0.11)	-0.12 (0.10)	0.04 (0.08)	0.08 (0.03)^b^	0.06 (0.03)^b^	0.17 (0.08)^b^	0.2 (0.13)
**constant**	-3.39 (0.32)^c^	-3.82 (0.32)^c^	-3.46 (0.11)^c^	-3.68 (0.10)^c^	-3.73 (0.19)^c^	-3.79 (0.22)^c^	-0.47 (0.19)^b^	-0.52 (0.15)^c^	-0.52 (0.06)^c^	-0.58 (0.06)^c^	-0.55 (0.55)^c^	-0.59 (0.16)^c^
**R**^**2** ($)^	0.27	0.36	0.33	0.33	0.38	0.37	0.78	0.82	0.79	0.77	0.73	0.66
**Obs**	2,814	2,546	24,932	27,103	8,321	6,138	2,814	2,546	24,932	27,103	8,321	6,138

^($)^: R^2^ referred to Pseudo R^2^ in the model for number of PHI-eligible services, and Adjusted R^2^ in the model for OOP for SHI eligible services

^a, b, c^ referred to 10%, 5%, 1% significance levels, respectively

Illness was a dominant factor causing a subsequent healthcare visits and excessive related OOP in all 3 groups. Illness induced a visit occurrence quite similarly across groups (Coef more or less than 4.30) yet costed more OOP of the high-income group than OOP of the middle- and low-income groups (Coef approximately 6.50, 6.00 and 5.00 in the high-, the middle- and the low-income groups, respectively). Generally, older ages, female, married, and not-working status were factors associated with higher incidence rate of eligible visits in the 3 groups. These factors also connected with higher OOP in the high- and the middle-income groups but not necessarily in the low-income group. For example, the age groups from 20–59 neither levied higher OOP nor mattered to OOP for these services in the low-income group. On the other hand, minor ethnicity was associated with lower incidence rates of visits as well as lower OOP, significantly in the low-income group (Coef -0.38 and -0.39 for the number of visits, and Coef of -0.17 and -0.26 for the OOP in 2014 and 2016, respectively).

#### Total health expense

The expense included healthcare charges for all kind of services, health insurance premium, drug and healthcare assisted device costs, if any, that a person had to pay. Result was presented in Table 4. As clearly seen, dominant factors increased the expense were sickness and maternity. Illness costed the middle-income’s OOP the most, followed by the high- and the low-income groups (Coef = 3.85, 3.58, and 2.97 in the middle-, the high-, and the low-income groups in 2016, respectively). Maternity costed more but with decreasing effects to the 3 groups, with the most expensive to the high-income, then less and less to the middle- and low-income groups (Coef = 5.89, 5.57, and 4.26 in the high-, the middle-, and the low-income group in 2016, respectively). Older ages and men were also obviously correlated with higher health expenses in all groups. Married status, education of the head of household and household income were positively linked to higher expense in the middle-income group, but not quite so in the high- and low-income groups. On the other hand, household size was negatively associated with total expense per person in all group. Minor ethnicity significantly reduced the expense in the low-income, but not the high-income group. Meanwhile residing in rurality decreased the expense in the high-income but not quite so in the middle- and low-income groups.

**Table 4:** The mechanisms of factors associated with total health expense in each household group

	**High-income group**		**Middle-income**		**Low-income**	
*Variable*	*Pre—2014*	*Post—2016*	*Pre—2014*	*Post—2016*	*Pre—2014*	*Post—2016*
	*Coef (se)*	*Coef (se)*	*Coef (se)*	*Coef (se)*	*Coef (se)*	*Coef (se)*
**Maternity**	6.25 (0.37)^c^	5.89 (0.45)^c^	5.49 (0.15)^c^	5.57 (0.15)^c^	4.61 (0.26)^c^	4.29 (0.33)^c^
**Illness**	3.56 (0.14)^c^	3.58 (0.15)^c^	3.87 (0.05)^c^	3.85 (0.05)^c^	3.52 (0.10)^c^	2.97 (0.15)^c^
***Age group (reference: Age 0—19)***						
Age 20—39	0.001 (0.25)	0.23 (0.27)	0.46 (0.07)^c^	0.43 (0.08)^c^	0.69 (0.13)^c^	0.57 (0.15)^c^
Age 40—59	1.64 (0.29)^c^	1.87 (0.30)^c^	1.90 (0.09)^c^	1.73 (0.09)^c^	1.66 (0.15)^c^	1.75 (0.18)^c^
Age 60 +	1.99 (0.32)^c^	2.14 (0.31)^c^	1.79 (0.09)^c^	1.64 (0.10)^c^	1.69 (0.15)^c^	1.82 (0.20)^c^
**Female**	-0.65 (0.14)^c^	-0.50 (0.15)^c^	-0.83 (0.04)^c^	-0.82 (0.04)^c^	-0.79 (0.07)^c^	-0.72 (0.09)^c^
**Minor ethnicity**	-0.65 (0.40)	-0.41 (0.26)	-0.14 (0.06)^b^	-0.12 (0.07)	-0.24 (0.07)^c^	-0.49 (0.11)^c^
**Married**	0.34 (0.18)^a^	0.23 (0.20)	0.42 (0.06)^c^	0.47 (0.07)^c^	0.2 (0.13)	0.02 (0.18)
***Employment status (reference: Being-employed)***						
Self-employed	-0.01 (0.16)	-0.16 (0.17)	-0.04 (0.06)	0.12 (0.06)^b^	-0.20 (0.10)^c^	0.01 (0.14)
Not employed	-0.06 (0.21)	-0.14 (0.25)	-0.08 (0.07)	0.14 (0.08)^a^	-0.53 (0.13)^c^	-0.53 (0.19)^c^
***Education of the household head (reference: primary school or lower)***						
Secondary or high school	0.19 (0.17)	0.03 (0.15)	0.12 (0.04)^c^	0.22 (0.04)^c^	0.19 (0.07)^c^	0.18 (0.11)
College or higher	0.26 (0.22)	0.43 (0.19)^b^	0.23 (0.10)^b^	0.28 (0.09)^c^	0.24 (0.34)	1.62 (0.81)^b^
**Household income (in ln)**	0.55 (0.21)^c^	0.28 (0.18)	0.17 (0.05)^c^	0.27 (0.06)^c^	0.25 (0.13)^a^	-0.14 (0.09)
**Household size**	-0.29 (0.04)^c^	-0.21 (0.05)^c^	-0.16 (0.01)^c^	-0.11 (0.02)^c^	-0.11 (0.02)^c^	-0.10 (0.03)^c^
**Rural residence**	-0.51 (0.15)^c^	-0.32 (0.15)^b^	-0.02 (0.05)	-0.09 (0.05)	0.12 (0.13)	0.38 (0.18)^b^
**constant**	3.06 (0.36)^c^	2.47 (0.36)^c^	1.80 (0.10)^c^	1.48 (0.11)^c^	1.80 (0.20)^c^	1.62 (0.26)^c^
**Adjusted R**^**2**^	0.39	0.38	0.45	0.42	0.44	0.38
**Obs**	2,814	2,546	24,932	27,103	8,321	6,138

^a, b, c^ referred to 10%, 5%, 1% significance levels, respectively

### The likelihood of falling into either the middle- or the low-income group

**Fig. 3:**
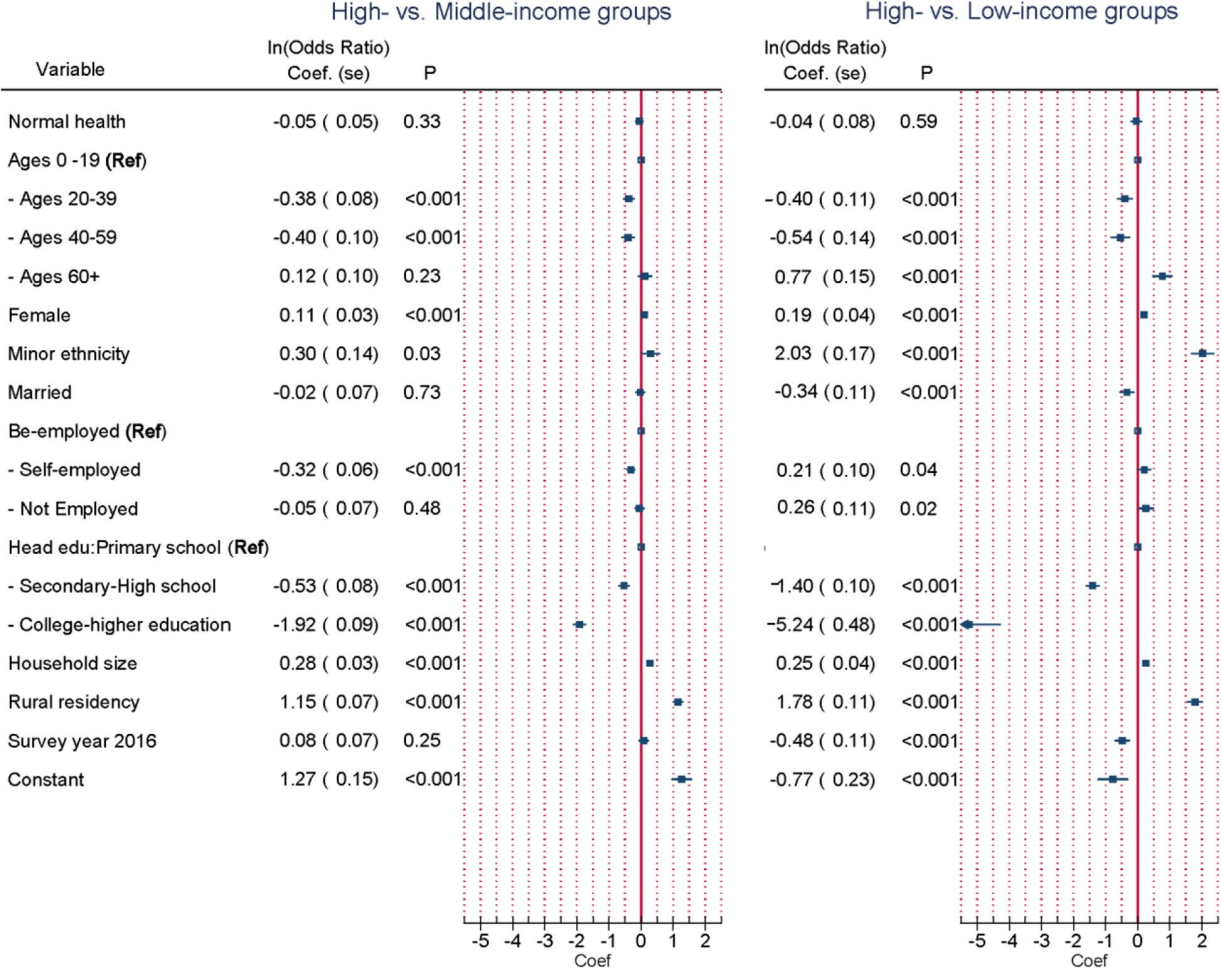
Factors associated with probability of falling into either the middle- or the low-income groups

This section depicted a mechanism which affected a likelihood of a person ‘s falling into either the middle-income or the low-income group. Pairwise comparisons between the high- and the middle-income group, and between the high- and the low-income groups were implemented and presented in their forest plots in Fig. 3.

The probability of a person being in the middle-income group instead of high-income would increase significantly if the person was of an ethnic minority (Coef = 0.30), female (Coef = 0.11), living in a rural area (Coef = 1.15), and in a bigger household size (Coef = 0.28). In contrast, a person seemed to be less likely in the middle-income group if the person was self-employed (Coef = -0.32), with a head of household having an education degree higher than primary school (Coef = -0.53 and -1.92 of secondary-high school and college-higher education, respectively), and at working age from 20 to 59 (Coef = 0.38 of ages from 20–39, and -0.40 ages 40–59). (Fig. 3, column “High- vs Middle-income groups”).

Similarly to the middle-income group, the probability of being in the low-income compared to the high-income group would increase if a person was either minor ethnicity (Coef = 2.03), female (Coef = 0.19), living in a rural area (Coef = 1.78), and in a bigger family (Coef = 0.25), with effects of minor ethnicity and rural residence outstandingly stronger than in the middle-income group. However, differently from the middle-income group, we observed that both self-employed (Coef = 0.21) and none-employed (Coef = 0.26) working status in this group contributed to bigger probability. Factors associated with less likelihood in the low-income group were higher education of the head of household (Coef = -1.40 and -5.24 for secondary-high school and college-higher education, respectively), at working ages from 20–59 (Coef = -0.40 and -0.54 for ages 20–39 and 40–59, respectively) and being married (Coef = -0.34). (See Fig. 3, column “High- vs Low-income groups).

### The estimated average treatment effects on the middle- and the low-income group

Table 5 displayed the results of the estimates ATT of the law change ${\widehat{\tau }}^{DR.DID}$ on the middle- and low-income households. The impacts on the middle-income group were significantly evidenced with an increase in PHI enrollments by 9%, and an increase in the number of visits for PHI-eligible services by 0.43 times per person in the post-amendment. For PHI beneficiaries in this group (subsample 1), the estimated impact on the number of visits was stronger, with an increase of 0.59 time per person (i.e., a household of 2 PHI beneficiaries would have approximately 1 more visit in the post-amendment than the pre-amendment period). However, in terms of OOP payments for PHI-eligible services and total health expenses, no significant changes were detected in both full sample and subsamples.

**Table 5:** ﻿Average effects of the law change on the middle- and the low-income groups

Outcome	Middle-income	Low-income
	ATT ${\widehat{\tau }}^{DR.RC}$	ATT ${\widehat{\tau }}^{DR.RC}$
***Full sample:***		
*PHI enrollment*	0.09^c^ (0.02)	0.08^a^ (0.047)
*Number of visits for PHI-eligible services*	0.43^b^ (0.18)	0.53 (0.51)
*OOP for PHI-eligible services*^€^	-0.01 (0.07)	-0.28 (0.21)
*Total health expense*^€^	0.06 (0.12)	0.06 (0.36)
No. of observations	57,395	19,819
***Subsample 1: only PHI beneficiaries***		
*Number of visits for PHI-eligible services*	0.59^b^ (0.21)	0.92^b^ (0.47)
*OOP for PHI-eligible services*	0.003 (0.09)	-0.28 (0.33)
*Total health expense*	0.02 (0.15)	-0.01 (0.5)
No. of observations	37,743	15,617
***Subsample 2: only people having at least a visits for PHI-eligible services***		
*OOP for PHI-eligible services*	-0.08 (0.18)	-0.83 (0.66)
*Total health expense*	0.03 (0.23)	-0.49 (0.68)
No. of observations	17,460	5,279
***Subsample 3: only SHI beneficiaries with at least a visit for PHI-eligible services***		
*OOP for PHI-eligible services*	-0.03 (0.23)	-0.73 (0.84)
*Total health expense*	-0.10 (0.28)	-0.57 (0.81)
No. of observations	12,616	4,253

^€^: OOP for PHI-eligible services and total health expense are in the natural logarithm of monetary payment

^a, b, c^ referred to 10%, 5%, 1% significance levels. Standard errors are in parentheses

Regarding the low-income group, the amendment increased PHI enrollments by 8% but did not significantly increase the number of PHI-eligible visits in this group. The OOP payments were not significantly reduced though lowering by approximately 28% in the post-amendment. If only PHI beneficiaries in this group were evaluated, higher PHI utilizations were evidenced with the visit number in the past 12 months increasing approximately by 1 more visit per person. Yet their OOP and total health expense showed insignificant reductions. Further examining persons who had at least one visit, the OOP and expenses also did not significantly decrease though their estimated differences between the pre and post-amendment were quite large, i.e., approximately by 56.4% and 51.8% for OOP payments, and 38.7% and 43.4% for total health expenditure in subsamples 2 and 3, respectively^6^ (section “Low-income” of Table 5).

## Discussion and conclusion

Our analysis showed that overall, the amendment played an important role in expanding PHI enrollments and utilizations but did not significantly reduce health expenditures for the middle- and low-income households during the 2014 – 2016 period. The pre-treatment survey 2014 showed that the middle-income group considered PHI as a necessary contingency service, and the low-income group, on contrary, considered PHI as an inferior contingency service. However, both of these groups turned into having nonsignificant income effects on PHI enrollment in the post-treatment survey 2016. These changes could be mainly resulted from the PHI expansion with extensive subsidies in premium and medical coverage for household members, especially in the low-income group.

There was a clear sign of health adverse selection with the strongest evidence in the middle-income group since persons with a normal health status were less likely to enroll into PHI than the others. This common problem in public health insurance was usually observed in voluntary insurance programs in many LMICs such as China, Philippine and Burkina Faso [30, 31] where people with a higher risk of being ill chose to enroll in health insurance yet healthy people decided not to sign up. Adverse selection can ruin the risk-pooling strategy and impoverish PHI financially as the publicly funded insurance must reimburse claims for medical costs from the illness at an increasingly large proportion of its revenue where the contribution by the healthy is relatively small. The amendment’s PHI expansion indeed mitigated this problem in 2016 by reducing the health adverse selection in the middle-income and alleviating it in the low-income group. It is predicted that once PHI covers the whole population this adverse selection will disappear. Future research, therefore, would focus on how this behavior changes along the pathway to UHC by 2030.

Furthermore, compared to those being-employed, the self-employed persons in the high- and middle-income group seemed to be less likely to enroll in PHI in the pre-treatment period 2014. This finding reflected challenges at that time to get these persons covered, and was consistent with the evidence in LMICs where PHI can easily enroll the poor and persons working in a formal sector but face incredible difficulties to cover the non-poor self-employed working in an informal sector [32]. However, some positive signs in motivating more enrollments by these self-employed persons were observed in the post-treatment 2016.

With regard to health service utilization, the amendment increased PHI utilization in terms of the number of healthcare visits by the middle-income and the poor and near-poor groups who were more likely to be an ethnic minority, female, living in a rural area, and having a bigger family size. These findings were consistent with other earlier research on the positive effect of public health insurance towards vulnerable and socially disadvantaged populations in Vietnam [9, 32] and in LMICs such as Peru, Indonesia and Colombia [33–35]. A possible explanation is the induced-demand effect of health insurance which encourages these groups – now possessed health insurance – to see doctors for common or minor health issues which otherwise they would not without health insurance.

However, the amendment so far did not significantly reduce OOP expenditures in both middle and low-income groups. This result contributed additional evidence that public health insurance did not considerably mitigate financial risks to low-income households in Vietnam [10, 36] and in other LMICs such as Ghana, Mexico and China [37–39]. It indicated big challenges in balancing high-quality care provision and full financial protection in Vietnam since most people, including the poor, prefer to seeking a better quality of care in high-level health facilities where are fully equipped with high medical technology, highly skilled doctors, nurses and healthcare staff [40].

Reducing OOP expenditures, therefore, requires not only PHI coverage expansions but also possible access to quality and affordable care. One major obstacle to the access during this period was certain bypassing-facility restrictions on PHI reimbursement that made those needs for quality care become unaffordable to many people. To address this issue, the amendment set to remove these restrictions on a specific timeline taking effect gradually where enrollees can bypass up to provincial-level hospitals for inpatient care/treatments without losing their full benefits. The approach was to reduce then eliminate the co-pay rates and allow full reimbursement in most cases of bypassing facilities.

Additionally, it is noteworthy that the amendment did not affect all income groups. The PHI enrollment rates in the high-income group were unchanged and still quite moderate (more or less 70%) during the study periods. A possible reason was that more affluent households might prefer to be enrolled in commercial insurance programs or be willing to pay their OOP for services in private providers [41]. The fact that the amendment did not regulate either PHI coverage with commercial insurance or PHI contracting relationships with private health providers had made PHI less attractive to these groups. According to a report in 2018 [42], approximately only 1.5% (500 out of more than 30,000) of private clinics, and 87.9% (160 out of 182) of private hospitals had contracts with PHI and received partial or full PHI reimbursement.

If PHI aimed to attain an universal health coverage, then further interventions should focus on getting this population more involved. Additional regulations and policies are needed to ease the participation of private clinics and health providers in PHI programs. Contracting, as an effective tool of the government to adjust service provisions [43], does not only stimulate PHI enrollments of the top income group but also ensure the quality and equity of health access to the whole population in Vietnam.

Our study is limited by the types of health services, and health expenses available on the surveys. We evaluate PHI utilizations for general maternal care and illness treatment services. Therefore, further research should focus on what specific medical services – for example, health services for chronic diseases that are covered by PHI – contribute most to healthcare utilization and expenditures. Besides, this study does not take into account non-medical costs such as transportation which could make the low-income households exposed to lower health utilization or higher health expenditures.

In conclusion, the amendment had influential impacts on motivating PHI enrollment, increasing PHI utilizations but not reducing OOP expenditures significantly in the middle- and low-come households in Vietnam during the 2014 – 2016 period. Furthermore, the amendment had merely effects on PHI enrollments of the high-income group. These findings underscore that a high proportion of PHI coverage does not automatically and immediately result in achieving financial protection. To strengthen the effectiveness of PHI on risk-pooling and financial protection, the government should remove certain barriers to quality health access that could prevent the middle- and low-income beneficiaries from receiving both quality and affordable care, as well as motivate high-income participants in PHI. Our findings provide additional evidence to policymakers that PHI could be further promoted as a moderator for healthcare quality, equity and financial protection in Vietnam, thereby being a helpful example for other LMICs who share common approaches to UHC and at a similar stage as Vietnam.

## Supplementary Information

**Additional file 1: Appendix 1. – **Examples of Healthcare Questionnaires. **Appendix 2.** – Tests for Conditional Parallel Trend Assumption (PTA) in the pre-amendment years, 2012 and 2014.

## Data Availability

The datasets used and/or analyzed during the current study available from the corresponding author on reasonable request;

## Footnotes

1 PHI typically covers eligible prescriptions and related medical procedures regulated by the Vietnam Ministry of Health. The wellness or general health exams are not covered by PHI. Except that some certain types of vaccination are free under the national vaccination program, vaccination is also not covered by PHI.

2 The survey does not cover institutionalized populations such as student housing, military compounds, hospitals, and prisons.

3 Household income per month per person.

4 The 2 steps of differencing gave the method the name of difference-in-differences.

5 A negative binomial regression can account for over-dispersion of the outcome, i.e., the dispersion is larger than the mean, by allowing a shape parameter. When the shape parameter is significantly different from 0, the negative binomial model is more suitable than the well-known Poisson model.

6 These effects in the low-income group were transformed from the ln(OOP) to a level change in OOP, i.e., e^(−0.83)^-1 = -0.564 for subsample 2; and e^−0.73^–1 = -0.518 for subsample 3.
