# Supplementary material for: How public health insurance expansion affects healthcare utilizations in middle and low-income households: an observational study from national cross-section surveys in Vietnam
Source: BMC Public Health. 2023 Mar 31;23:624. doi: 10.1186/s12889-023-15500-6 (PMC10067245; doi:10.1186/s12889-023-15500-6)
Supplement: Supplementary file 1 — Additional file 1: Appendix 1. – Examples of Healthcare Questionnaires. Appendix 2. – Tests for Conditional Parallel Trend Assumption (PTA) in the pre-amendment years, 2012 and 2014. [file 12889_2023_15500_MOESM1_ESM.docx]

# **Appendices**

**Appendix 1 – Examples of Healthcare Questionnaires**

**1.** Over the last 12 months have you had such a severe injury that has to lie down in a place and be taken care by a bedside caregiver or stop working/studying/not participate in normal activities?

🞎 Yes, please answer the questions 1.1,1.2 🞎 No

1.1. How many times have you had such a severe injury over the last 12 months?

1.2. How many days have you had such a severe injury over the last 12 months?

**2**. Over the last 12 months, has anyone in your household visited medical establishments or had home visits by physicians for

🞎 health check-ups

🞎 Vaccination

🞎 pregnancy checks, abortion, insertion of intrauterine device, birth delivery in case of no sickness/diseases/injuries

🞎 Other medical treatment

**3.** Number of visits and costs for health checks/non-resident treatment over the past 12 months (costs include fees for check-ups, treatment, medicines, allowances for physicians, travel, purchase of facilities,... relating to visits for check-up/treatment)

**4**. Number and costs of visits for resident treatment over the past 12 months? Costs include hospital fees and others (allowances for physicians, charges for on-demand services, purchase of additional medicines, facilities, travel for caring,...) relating to the visits for check-ups/treatment)

**5.** Did the household afford the medical check/treatment for the ill person/people?

🞎 Yes 🞎 No

**6**. If the household did not afford, did you have to sell assets, withdraw savings or other means to pay the cost?

**7**. Over the past 12 months, have you had a health insurance card or a free healthcare booklet/card/certificate?

🞎 Yes 🞎 No

**8**. What type of health insurance do you have?

🞎 Booklet/card for children aged 6 or under

🞎 Health insurance card for the poor

🞎 Health insurance card for the near-poor

🞎 Free healthcare booklet/card/certificate

🞎 Health insurance card for policy beneficiaries

🞎 Other compulsory state-run health insurance card

🞎 Other compulsory non-state health insurance card

🞎 Voluntary health insurance card for students

🞎 Other voluntary health insurance card

🞎 Others, specify

**9**. How much have you spent on health insurance premiums over the past 12 months?

**10**. What type of services you used the health insurance cards or free healthcare booklets/cards/certificates during visits over the past 12 months?

🞎 Outpatient 🞎 Inpatient

**11**. How much has your household spent on purchasing medicines without check-ups (prescriptions) for self-treatment or reserves over the past 12 months? (including expenditures on medicines and others, such as travel, vehicle-depositing fees,...)

**12**. How much has your household spent on purchasing medical materials over the last 12 months? For instance, stethoscopes, blood pressure monitors, hearing aids, phlegm absorbers, medicine cabinet, clinical thermometers, cotton, bandage, compresses...?

**Note**: All household members are identified by their age, sex, marital status, working status, educational levels and ethnic.

**Appendix 2 – Tests for Conditional Parallel Trend Assumption (PTA) in the pre-amendment years, 2012 and 2014**

|  | **Middle-income** | **Low-income** |
| --- | --- | --- |
|  | PTA $\hat{\tau}^{DR.RC}$ (s.e.) | PTA $\hat{\tau}^{DR.RC}$ (s.e.) |
| *Full sample* |  |  |
| SHI enrollment | -0.037 (0.023) | 0.001 (0.06) |
| No. of visits for SHI-eligible services | -0.28 (0.25) | -0.62 (0.86) |
| ln(OOP for SHI-eligible services) | -0.02 (0.07) | 0.15 (0.16) |
| ln(total health expense) | 0.07 (0.12) | 0.03 (0.37) |
| No. of observations | 52,685 | 25,420 |
| *Subsample 1: including SHI beneficiaries* | | |
| No. of visits for SHI-eligible services | -0.45 (0.29) | -1.09 (0.68) |
| ln(OOP for SHI-eligible services) | -0.05 (0.11) | 0.17 (0.26) |
| ln(total health expense) | -0.02 (0.16) | 0.19 (0.52) |
| No. of observations | 31,285 | 18,663 |
| *Subsample 2: including those having at least one visits for SHI-eligible services within 12 months* | | |
| ln(OOP for SHI-eligible services) | -0.02 (0.17) | 0.38 (0.5) |
| ln(total health expense) | -0.2 (0.21) | -0.04 (0.78) |
| No. of observations | 16,417 | 6,953 |
| *Subsample 3: including SHI beneficiaries with at least one visit for SHI-eligible services* | | |
| ln(OOP for SHI-eligible services) | -0.03 (0.3) | 0.46 (0.84) |
| ln(total health expense) | -0.2 (0.29) | 0.06 (1.12) |
| No. of observations | 10,846 | 5,172 |

(1): PTA $\hat{\tau}^{DR.DID}$estimated using the pair of middle-income and high-income groups during 2 pre-amendment periods, 2012 and 2014. In the absence of amendment’s effect on the middle-income group, if PTA holds then PTA $\hat{\tau}^{DR.DID}$ should be not significantly different from 0.
(2): PTA $\hat{\tau}^{DR.DID}$estimated using the pair of low-income and high-income groups during 2 pre-amendment periods, 2012 and 2014.
^(*)^, ^(**)^, ^(***)^: 10%, 5%, 1% significance levels. Standard error of the estimate is in parentheses.
